# Important Ruling: Powers of Boards of Health

**Published:** 1908-05

**Authors:** 


IMPORTANT RULING: POWERS OF BOARDS OF HEALTH.—Unincorporated towns and villages may have boards of health and these boards can promulgate rules and regulations and enforce them. James D. Walthall, Office Assistant Attorney General, has rendered an opinion to the State Health Department in which it is held that boards of health in unincorporated towns and villages have the power to promulgate rules and regulations for the public health, which rules and regulations shall be obeyed as laws, and such boards shall have the power to enforce them. In this connection, State Health Officer Brumby is now preparing a code of rules and regulations for such unincorporated districts, and is also making an effort to have these towns and villages incorporate for sanitary purposes. In the opinion Mr. Walthall says:

"This Department is in receipt of your inquiry concerning the powers and duties of boards of health for unincorporated towns and villages in this State. We think such boards have power to make regulations and orders of general application which shall be published and obeyed as laws; also to make special orders, to meet emergencies not provided for by regulations of general obligation, to be enforced for the suppression of nuisances and, of course, of contagious diseases and other great dangers to life and health.

"The duties which commonly devolve upon such boards may be enumerated as follows:

"First—To discover and remove preventable causes of disease, and to this end to maintain an efficient system of sanitary inspection and reports.

"Second—To appoint agents and inspectors to carry into effect the sanitary rules and regulations.

"Third—To cause every case of infectious or contagious disease to be reported immediately to the board.

"Fourth—To keep the State Health Officer informed concerning dangerous diseases in its jurisdiction.

"Fifth—To receive and examine into the nature of complaints concerning nuisances and causes of danger and injury to public health and to cause such nuisances and all other things dangerous to the public health to be removed, suppressed and abated.

"Sixth—To protect the community and every family against infectious and epidemic diseases, and to prevent the spread of contagion by providing for the regular isolation of domestic quarantine of every case of contagious disease which occurs in the community.

"The powers are conferred and the duties enjoined upon the board to secure the preservation and promotion of the public health by the enforcement of all necessary and proper sanitary regulations and the suppression, amendment or removal of all conditions detrimental to the lives and health of the people. And in view of the importance of the interest confided to the care of such health boards the laws conferring those powers receive a liberal construction in aid to the beneficial purposes for their enactment.

"In the case of Gregory vs. New York, 40 New York, 275, the court say: 'The importance of sustaining local boards of health in all lawful measures tending to secure or promote the public health should make the courts cautious in declaring any curtailment of their authority, except upon clear grounds.'"

This applies to cities as well. It may be asked how can the health board enforce its mandates? Dr. Brumby, the State Health Officer of Texas, under and by authority of the above ruling, compelled two slaughter houses in Austin to discontinue, to build new houses on clean ground, to tear down and burn the old buildings and cover the entire ground with sand and lime. How did he do it? By threat of injunction and suit for maintaining a public nuisance—a menace to the public health. Bully for Brumby!

